# From Molecules to Amoeboid Movement: A New Way for Understanding the Morphology Through Actin-Binding Proteins

**DOI:** 10.3390/biom14121583

**Published:** 2024-12-11

**Authors:** Ekaterina Volkova, Igor Pozdnyakov, Mikhail Petukhov, Valeriia Polezhaeva

**Affiliations:** 1 Zoological Institute RAS, St. Petersburg 199034, Russia; igor.pozdnyakov@zin.ru (I.P.); valeria.polezhaeva@zin.ru (V.P.); 2 Petersburg Nuclear Physics Institute, NRC “Kurchatov Institute”, Gatchina 188300, Russia; petukhov_mg@pnpi.nrcki.ru; 3 Institute of Applied Computer Science, ITMO University, St. Petersburg 197101, Russia

**Keywords:** Arp2/3, free binding energy, Amoebozoa, amoeboid movement, hyaloplasm

## Abstract

Amoebozoa is a group of single-celled organisms that change their shape during locomotion. However, there is a taxon-specific complex of morphological characters inherent in the moving amoebae, known as locomotive forms. Actin is one of the proteins most important for amoeboid movement that, together with actin-binding proteins, construct the architecture of the cytoskeleton in the amoeboid cells. One of the actin-binding proteins is the Arp2/3 complex that provides a connection between actin filaments at an angle of 70°. In this paper, we predicted 3D models of bonded subunits Arp2 and Arp3 for 30 species from different taxa of Amoebozoa based on the publicly available transcriptomic data. Moreover, we predicted the binding free energy (ΔG) of bonded subunits Arp2 and Arp3 for 30 species and tried to link it to the morphology of the locomotive forms of amoebae. The ΔG values are the lowest in amoebae with the broad hyaline area, like *Vannella* spp. Amoebae that produce thin hyaline projections, like *Vexillifera abyssalis*, are characterized by intermediate ΔG values. Finally, the highest ΔG values are typical for the group of amoebae that have no conspicuous hyaline areas of the cytoplasm, like *Pelomyxa shiedti*, or have small hyaline caps, like *Arcella intermedia*. The presented analysis provides new insights into the molecular mechanisms of shape formation in amoeboid cells.

## 1. Introduction

Amoeboid organisms do not possess a constant cell shape, and their activities, such as locomotion or feeding, rely on cell shape changes and cytoplasmic protrusions. Amoeboid cells are present in different lineages of eukaryotes, but there are several lineages, comprising mainly amoebae. Amoebozoa, branching close to the common root of Opisthokonta, are one of these lineages [1]. The locomotive form of Amoebozoa comprises the major set of morphological taxonomic characters in these organisms [2,3]. It is mainly characterized by the outline and cross-section of the moving cell, spatial organization of hyaloplasm and granuloplasm, type of cytoplasmic projections, and shape of uroid and dorsal ridges or folds. For the comprehensive designation of the morphological characters of the locomotive form, the term “morphotype” was introduced. The morphotype is a general pattern of the morphodynamic organization of a locomotive form of an amoeba [3]. The authors initially distinguished 19 morphotypes of Amoebozoa [3]. In general, the morphotypes are distributed between two major clades in Amoebozoa: Discosea are flattened in the cross-section and have a polyaxial flow of cytoplasm, and Tubulinea with a cylindrical cross-section and monoaxial cytoplasmic flow [4]. The consensus is that the features of morphotypes are provided with the dynamics of a cytoskeleton [5]. The microtubular cytoskeleton was revealed with immunofluorescence in most groups of Amoebozoa: Archamoebea, Eumycetozoa, Variosea, Cutosea, Centramoebida, and Vannellida [5,6]. The localization of actin microfilaments was shown for Archamoebea, Eumycetozoa, Cutosea, and Centramoebida [5,6,7,8,9]. In Amoebozoa, cytoplasmic microtubules are present in the granuloplasm, the part of cytoplasm containing inclusions, while the actin cytoskeleton is localized all through the cytoplasm, but mainly at the periphery of the cell, in the frontal hyaloplasm and subpsedopodia [5,6,8]. However, the microtubules are not an essential part of the cytoskeleton. There is an example of the absence of cytoplasmic microtubules in *Entamoeba histolytica* (Amoebozoa, Archamoeba) [7].

The actin cytoskeleton is an important element necessary for cell motility, feeding, adhesion, and other cell activity [10,11,12,13]. Along with actin, myosin plays a huge role in cell motility, especially in amoeboid movement, providing the contractility of the filaments binding with myosin. Based on previous studies, we can conclude that, in the frontal area of the leading edge of the migrating cell, there is no myosin [14,15,16,17,18,19]. Apparently, myosin is more required in the granuloplasmicarea containing inclusions. However, the presence of myosin I able to interact with the plasma membrane was detected for the cortical layer of *Acanthamoeba castellanii* [20]. In total, actin, myosin, and other actin-binding proteins, along with tubulin, regulate and maintain the architecture of the cytoskeleton. The hyaline area at the leading edge is one of the main features that determine the taxon-specific morphology of locomotive forms of amoebae—different kinds of subpseudopodia of Discosea (filopodia, acanthopodia, dactylopodia, etc.), the fronto-lateral area of Vannellida similar to lamellipodia, and the hyaline cap at the tip of the true pseudopodia of Tubulinea [3,21]. Since the hyaline zone of the locomotive form is a significant feature, we decided to pay attention to the Arp2/3 complex, one of the main factors that regulate the formation of hyaline structures [9,19,22].

Actin filaments can be organized into actin networks or bundles with actin-binding proteins (ABPs) [23,24,25]. One of the crucial ABPs is the Arp2/3 complex which plays an important role in cell motility, phagocytosis, and substrate adhesion [9]. The Arp2/3 was first identified in *Acanthamoeba castellanii* [26]. Later, the Arp 2/3 was discovered in most eukaryotic organisms, excluding some Chromalveolates and plants [27]. The complex provides a junction between the mother and newly polymerized actin filaments at the angle of 70° between them, making an actin network [28]. The Arp2/3 complex consists of seven subunits: Arp2, Arp3, and ArpC1–5 [29] (Pizarro-Cerdá, 2017). The Arp2 and Arp3 interact with monomers of actin directly, while the rest of the complex plays the role of a connection between the complex and the mother actin filament [29,30,31]. The Arp2/3 itself is inactive and unable to connect with actin monomers [32]. After activation with nucleation promoting factors (NPFs), the complex changes its conformation and the actin-binding sites become available for interaction with actin [31,33]. Previous research demonstrated the sites and amino acids playing a role in the interaction between Arp2, Arp3, and actin monomers in *Schizosaccharomyces pombe* [31] (Table 1).

The interaction between proteins in the complex affects the complex free binding energy. This interaction is expressed in the free energy of the complex and can be predicted with the molecular dynamic simulation. There is software such as Rossetta, Amber, and Gromacs that calculate the stability of a protein, position of the protons and water bridges, and the free energy of a complex formation based on the 3D structure [34,35,36]. In this way, the interactions between the subunits of the ARP2/3 complex and interactions between the AP2/3 complex and the mother/daughter filaments were studied [31,34]). The same software was used to predict interactions between the ARP2/3 complex and its inhibitors such as arpin and GMF [35,36]. However, the molecular interaction between subunits Arp2 and Arp3 was not considered in detail in the recent studies [31,34].

In this study, for the first time, we obtained primary data showing the relation between the functional features of the Arp2/3 complex and the morphology of amoebae. Since the Arp2 and Arp3 subunits interact directly with the actin monomers, we decided to focus on the interaction of these two subunits. We found out the interrelation between the amino acid sequences of the subunits Arp2 and Arp3, the free binding energy of the bonded subunits in an inactivated complex, and the morphology of 29 species of Amoebozoa. We also paid attention to the amino acid residues participating in the connection of Arp2 and Arp3 and revealed the taxon-specific sites in the sequences of Arp2 and Arp3 from Amoebozoa. Finally, we present the free binding energy of Arp2 and Arp3 for Amoebozoa based on molecular dynamics. By estimating the stability of the subunits binding to each other in the complex, we hypothesize the possible effect of the FBE on the morphological features.

## 2. Material and Methods

### 2.1. The Collection, Preparation, and Alignment of Sequences

For analyses, all sequences of ARP2 and ARP3 subunits of Amoebozoa were selected from the NCBI databases by the BLAST search system [37]. Protein sequences were selected by searching psi-BLASTp from the nr database. The queries were the ARP3 and ARP2 sequences of *Schizosaccharomyces pombe* (accession numbers in the NCBI Protein database 6W17_A and 6W17_B, respectively). The mRNA sequences were selected by searching tBLASTn in the refseq_rna and tsa databases with the same queries. The target sequences from the protein and nucleotide databases were obtained with a threshold score above 400. This threshold was defined based on the annotated sequences. Subsequently, the mRNAs were translated into amino acids with TransDecoder 5.5.0 (TransDecoder 5.5.0, n.d.). Finally, originally, protein sequences and sequences translated from mRNA were aligned with the Cobalt aligner (Papadopoulos and Agarwala). Sequences covering the query sequence by less than 75% were discarded as potential assembly errors or non-functional paralogs. In this way, separate alignments for Arp2 and Arp3 were obtained. The Arp2 (XP_645275.1) and Arp3 (XP_638880.1) sequences of *Dictyostellium discoideum* were chosen as references for a designation of amino acid positions. All alignment analyses were performed with SeaView tools, with manual correction based on common Arp2 and Arp3 multi-alignments of Amoebozoa.

### 2.2. The Prediction of 3D Models

The separate species set was prepared for 3D modeling. For every genus, a single species was selected. Two species were selected from each of the genera *Dictyostelium* and *Entamoeba* due to the availability of sequences from several species in these genera. The selection criteria were the quality of the genome assembly and the completeness of its annotation. The resulting set of 30 Amoebozoa species was used for 3D modeling. Three-dimensional models of the Arp2 connected to the Arp3 subunits of Amoebozoa were predicted based on the three-dimensional model of the complete Arp2/3 complex of *S. pombe* [31] by the Bioluminate 3:1 program of the Schrödinger 2020 package [38] (Bioluminate, n.d.). The 3D model of the protein complex of *S. pombe* 6W18 was downloaded from the Protein Data Bank (n.d.). The 3D models of Arp2 and Arp3 were extracted separately from the model of the whole complex with the help of Bioluminate. The published 3D structure of Arp2 and Arp3 from *S. pombe* had gaps in the sequences. To replenish these gaps, the 3D models were superimposed on their sequences in Swiss Model [39] (Swiss-Model, n.d.). Thus, the 3D models were completely replenished and saved as pdb files. To obtain the 3D models of the Arp2 and Arp3 for Amoebozoa species, the 3D models of *S. pombe* sequences were used as templates. For modeling of 3D structures, the “Advanced Homology Modeling” operation in the Bioluminate 3:1 software, with the help of “Prime STA” and “Energy Base” methods, was used. The resulting PDB files were regularized in MSI [40]. Then, the regularized PDB files underwent optimization and energy minimization with Bioluminate using the Protein Preprocessing Wizard. Finally, a model of Arp2 bound to Arp3 was compiled, followed by the optimization and energy minimization with Bioluminate. The function “Protein Interaction Analysis” predicted groups of interacting residues from one and other subunits and described the type of their interaction. Based on these data for each species, a summary table was constructed (Table S1). Moreover, visualizations of the inactivated Arp2 bound to Arp3 structure and the interaction sites of the subunits were obtained with Bioluminate.

### 2.3. Molecular Dynamics and Binding Free Energy Calculations

For the set of 30 Amoebozoan species, the free binding energy of the subunit Arp2 bound with the Arp3 was calculated. The binding energy of the connected subunits in the complex was calculated as a difference between free energy (ΔG) of the complex and unbound subunits. For calculation, the solvation effect was taken into account. All calculation were performed by the MM-GBSA method based on the results of molecular dynamics simulations in the Amber20 package [41] (The Amber Home Page, n.d., https://ambermd.org/). For the calculations, we used the ff14SB force field. The complex was placed in an octahedral box filled with water (TIP3PBOX water model). The minimum distance from the protein molecule to the box boundary was at least 12 Å. The system was prepared for molecular dynamics simulations through the following steps: (1) energy minimization (100 cycles of 1 fs; constant volume; the non-bonded cutoff is 8 Å), (2) heating (25,000 cycles of 2 fs; constant volume; cutoff is 8 Å; initial temperature is 100K; final temperature is 300 K; using a Langevin thermostat; the collision frequency gamma is set to 2 ps), (3) density equilibration (25000 cycles of 2 fs; constant pressure; isotropic scale; pressure relaxation time is 1 ps; cutoff is 8 Å; temperature is 300 K; using Langevin thermostat; the gamma is 2 ps), and (4) pressure equilibration (250,000 cycles of 2 fs; constant pressure; isotropic scale; pressure relaxation time is 2 ps; cutoff is 8 Å; temperature is 300 K; using Langevin thermostat; the gamma is 2 ps). The molecular dynamics simulation was performed in 4 stages under the same conditions as in the pressure equilibrating stage. Each stage included 2,500,000 cycles of 2 fs. Atomic coordinates were recorded every 5000 cycles. The total time for calculating the molecular dynamics trajectories was 20 ns. The calculations for preparing the system and simulating the molecular dynamics were performed on the GPU (NVIDIA GTX-1080TI). The Gibbs free energy estimation using the MM-GBSA method was performed on the CPU using the MMPBSA.py script (MMPBSA.py.MPI, a version for parallel computing). For *Entamoeba* species (parasites of mammals), additional ΔG calculations were made at 310 K. For the psychrophilic species *Vermistella antarctica* (lives at 0 °C), additional ΔG calculations were made at 273 K.

### 2.4. Statistical Analysis

To clarify whether the ΔG values have an effect on morphological features of locomotive forms of Amoebozoa or not, the ANOVA was performed. Amoebozoa species with a well-studied morphology were divided into four groups according to the presence/absence hyaloplasm: A (no hyaloplasm or hyaline cap), B (hyaline projections), C (hyaline projections and hyaline area), and D (hyaline projections and hyaline area). Some species of the Dictyostellids group, for which there were no data on the morphology of the amoeboid stage (myxamoebae), we discarded from the analysis. Thus, 21 species out of 30 with their ΔG values were included in the analysis. The ANOVA was performed with Excel 2016 followed by the Tukey’s test to assess the statistical significance of differences between the groups (Microsoft Excel 2016. Retrieved from https://office.microsoft.com/excel, accessed on 20 March 2023).

## 3. Results

### 3.1. Actin-Binding Sites of Amoebozoan Subunits Arp2 and Arp3

The amino acid alignment of ARP3 consists of 79 sequences including 30 genera of Amoebozoa and one reference sequence of *S. pombe* (Supplementary, S1). The amino acid alignment of ARP2 consists of 98 sequences including 28 genera of Amoebozoa and one reference sequence of *Schizosaccharomyces pombe* (Supplementary, S2). Based on the variability, we divided the amino acid residues of Arp2 and Arp3 into three groups:

-- identical (Ident)—the residue is identical in all studied species of Amoebozoa;
-- conservative (Cons)—the residue is identical to the residue from reference sequences (XP_638880, XP_645275) or is substituted by a residue from the group of amino acids with the same properties;
-- variable (Var)—the residues vary among different species of Amoebozoa (Supplementary, Table S1).

**Figure 1:**
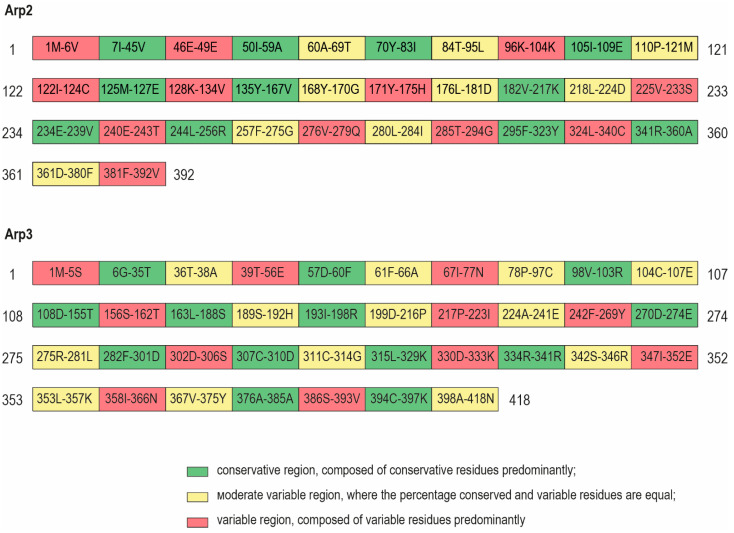
The sequences regions of Arp2 and Arp3 separated by their variability.

Three types of regions are distinguished along the sequences according to the saturation of conservative residues:

-- conservative (Cons)—a region composed of conservative residues predominantly;
-- moderate variable (Mod var)—a region of a molecule where the percentage conserved and variable residues are equal;
-- variable (Var)—a region composed of variable residues predominantly (Figure 1).

We selected four amino acids that were shown as important amino acids for the actin–ARP2 interaction and six amino acids for the actin–ARP3 interaction in *Schizosaccharomyces pombe* 6W17 (Table 1). The Amoebozoan sequences were compared with 6W17. We revealed only one conservative position for Arp2 and three conservative positions for Arp3. Other amino acids differed from those of *S. pombe* in all or some of the Amoebozoan species compared. The revealed conservative AA and observed differences are presented in Table 2.

### 3.2. 3D Models and AA Residues Playing Role in the Interaction Between Subunits

**Figure 2:**
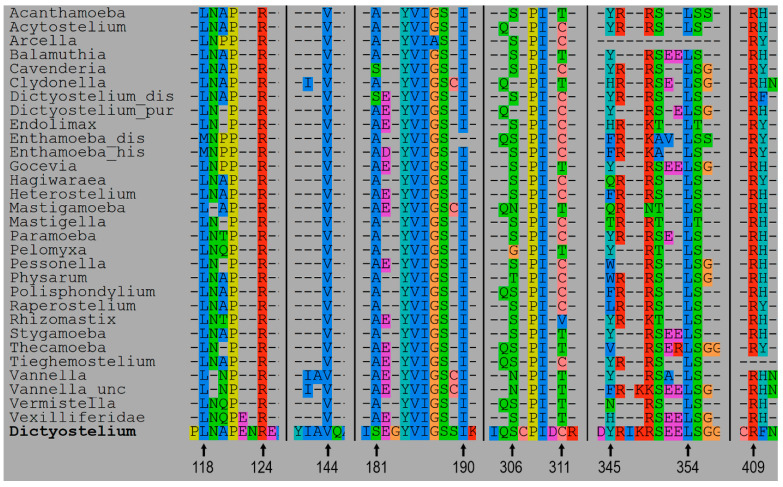
The multi-alignment of Arp3 amino acid residues involved in the Arp3–Arp2 interaction on the *D. discoiceum* full Arp3 sequence. Amoebozoan species: Acanthamoeba—*A. castellanii*, Acytostelium—*A. subglobosum*, Arcella—*A. intermedia*, Balamuthia—*B. mandrillaris*, Cavenderia—*C. fasciculata*, Clydonella—*C. sp.*, Dictyostelium_dis—*D. discoideum*, Dictyostelium_pur—*D. purpureum*, Endolimax—*E.* sp., Enthamoeba_dis—*E. dispar*, Enthamoeba_his—*E. histolytica*, Gocevia—*G. fonbrunei*, Hagiwaraea—*H. rhizopodium*, Heterostelium—*H. album*, Mastigamoeba—*M. abducta*, Mastigella—*M. eilhardi*, Paramoeba—*P. aestuarina*, Pelomyxa—*P. schiedti*, Armaparvus—*A.* sp., Physarum—*P. polycephalum*, Polisphondylium—*P. pallidum*, Raperostelium—*R. potamoides*, Rhizomastix—*R. vacuolata*, Stygamoeba—*S. regulate*, Thecamoeba—*T. quadrilineata*, Tieghemostelium—*T. lacteum*, Vannella—*Vannella* sp., Vannella_unc_—*V.* sp. uncultured, Vermistella—*V. antarctica*, Vexilliferidae—*V. abyssalis*. Arrows show residue position on *D. discoideum* sequence. Black vertical lines separate the regions of interaction.

**Figure 3:**
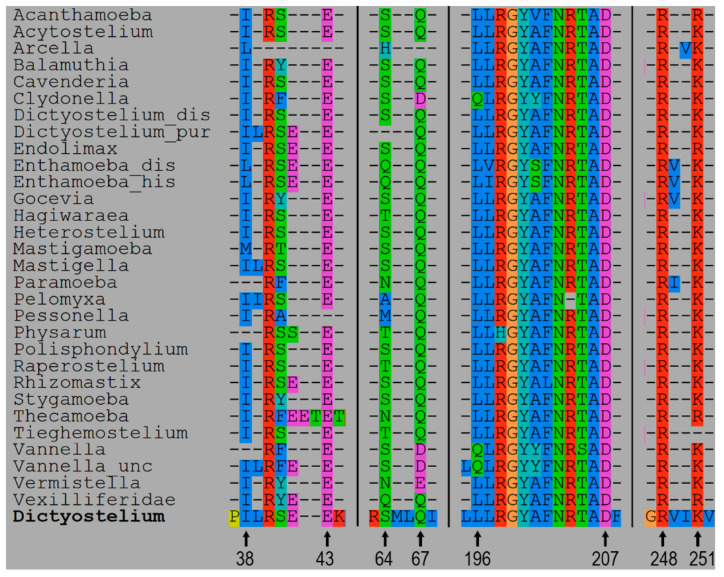
The multi-alignment of Arp2 aminoacid residues involved in the Arp3–Arp2 interaction on the *D. discoiceum* full Arp2 sequence. The designations are same as in Figure 2.

**Figure 4:**
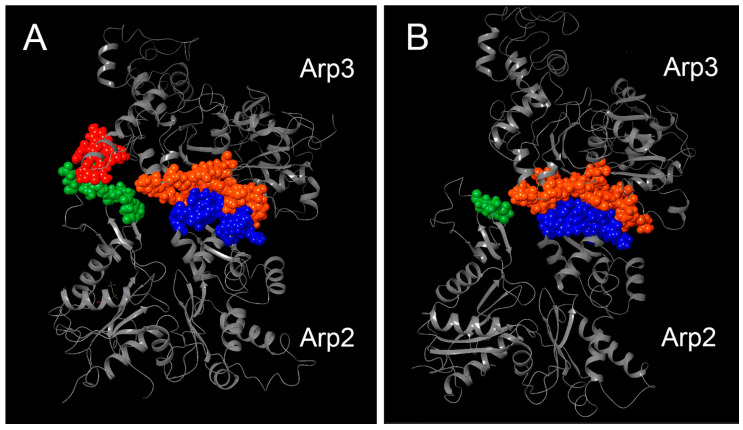
The 3D models of Arp2 binding with Arp3 of Amoebozoa. (**A**) 3D model of Arp2 binding with Arp3 of *Dictyostellium doscoideum*. The first site of interaction is colored orange and blue in Arp3 and Arp2, respectively. The second site of interaction is colored red and green colors in Arp3 and Arp2, respectively. (**B**) 3D model of ARP2 binding with Arp3 of *Arcella intermedia*.

**Figure 5:**
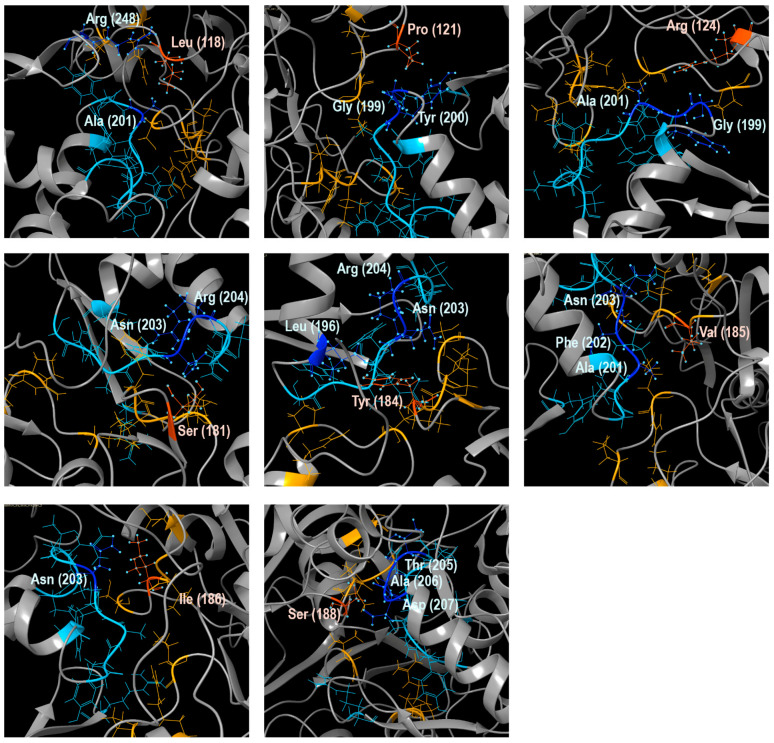
Visualization of the spatial interaction of amino acids between the subunits Arp3 and Arp2. Several interacted amino acids of the Arp3 and Arp2 sequences are shown at an atomic level and colored in dark orange and dark blue, respectively. The full regions of interaction in Arp3 and Arp2 are colored in light orange and light blue.

For every species, the 3D structures of the unbound and connected subunits Arp2 and Arp3 in the inactive form were predicted (Supplementary, S3). Based on these models, the amino acid residues interacted between subunits Arp2 and Arp3 and the type of their interaction were identified for all species (Table 3; Supplementary, S4). The amino acid residues of Arp2 and Arp3 involved in interactions between the subunits were extracted as separate sets of amino acids for every species of Amoebozoa. These amino acid sets from Arp2 and Arp3 were aligned to the full sequences of Arp2 and Arp3, respectively (Figure 2 and Figure 3). We present and describe in detail below the 3D model of the interacting subunits of *Dictyostelium discoideum* (XP_638880, XP_645275) as an example (Table 3, Figure 4A). According to the analysis of protein interactions, there is van der Waals surface complementarity for 15 amino acids of the interacting amino acid residues. There is no vdW complementarity between the rest of the interacting residues. Despite the presence or absence of vdW complementarity, a “Buried SASA” (a fraction of the solvent-accessible surface area of the residue that, due to the proximity with residues in the other group, became buried and inaccessible to the solvent) is always present (Table 3). We revealed two sites of connection between the subunits presented by four regions in the subunits (Figure 4A). For convenience, the regions were colored in pairs of interaction in the 3D model: orange and blue regions of Arp3, and red and green regions of Arp2 (Figure 4A). In *D*. *discoideum*, the first site of interaction was formed by the region in Arp3 L(118)-P(121)-R(124)-V(144)-S(181)-E(182)-Y(184)-V(185)-I(186)-G(187)-S(188)-I(190)-S(306)-P(308)-I(309)-C(311)-S(406)-R(409)-F(410)-I(414)-I(417) that sterically interacted with the region L(196)-L(197)-R(198)-G(199)-Y(200)-A(201)-F(202)-N(203)-R(204)-T(205)-A(206)-D(207)-R(248)-K(251) in Arp2 (Figure 1, Figure 2 and Figure 3). The second site of interaction consisted of the region Y(345)-R(346)-R(349)-S(350)-L(353)-S(354) in Arp3, that sterically interacted with the region I(38)-R(40)-S(41)-E(43)-S(64)-Q(67) in Arp2 (Figure 2, Figure 3 and Figure 4A). Despite the fact that the regions were sterically located together in the tertiary structure model of the subunit, they were discontinuous in alignment. Moreover, the second site of interaction of Arp3 Y(345)-S(354) is nested to the first site of Arp3 (Figure 2 and Figure 4A). The region in the Arp3 colored in orange in the 3D model is presented by five subregions: L(118)-R(124), V(144), S(181)-I(190), S(306)-C(311), and S(406)-I(417) (Figure 2 and Figure 4A). In turn, this region interacted with the corresponding one in the Arp2 colored blue (Figure 1). This region consists of three subregions: L(196)-D(207), R(248), and K(251) (Figure 3 and Figure 4A). The region in the Arp3 colored red in the 3D model consisted of subregions located closely to each other: Y(345)-R(346), R(349)-S(350), and L(353)-S(354) (Figure 2 and Figure 4A). This region interacted with the corresponding one in the Arp2 colored green that is presented by two subregions: I(38)-E(43) and S(64), Q(67) (Figure 3 and Figure 4A,). The interaction between amino acids is shown spatially using three subregions of the first site of interaction (Figure 5).The sites of interactions with their regions are very similar in all species of Amoebozoa considered in this study, excepting *Arcella intermedia.* There was a deletion of the region of Arp3 corresponding to Y(345)-R(346)-R(349)-S(350)-L(353)-S(354) in *D*. *discoideum* and there were unique substitutions at the region of Arp2 (Figure 3 and Figure 4B). These changes led to the loss of one of the interaction sites (Figure 2 and Figure 4B). For all Amoebozoan species, we provide a summary table showing the following:

-- interacting amino acids residues of *D. discoideum* that have vdW surface complementarity;
-- substitutions of these residues in other Amoebozoan species;
-- the ranges of vdW surface complementarity and Buried SASA between residues among Amebozoa (Supplementary, Table S2).

There are interacting identical amino acid residues identical for all Amoebozoa, i.e., Ile (186)-Asn (203). Despite their identity, there is a variability of surface complementarity within 0.55–0.85 and Buried SASA within 78.7–89.2% provided by the interaction of Ile (186)-Asn (203) (Supplementary, Table S2).

### 3.3. Free Binding Energy (∆G) of the Subunits Arp2 and Arp3 of Amoebozoa

The ∆G values of the subunit ARP2 binding to the ARP3 were calculated based on the 3D models. The ∆G at 27 °C varied from −58.8 to −28.6 kcal⋅mol^−1^ (average −42.5, SE = 0.28). The lowest ∆G was calculated for Vannellids (−58.8 to −52.4 kcal⋅mol^−1^) (Table 4). Energy values of other Amoebozoans did not correspond to their distribution among the taxa (Table 4). Dictyostelids, archamoebids, and centramoebids spread throughout the whole range of their ∆G values. Since several of the analyzed species may inhabit specific temperature conditions, the ∆G at 37 °C for parasitic species and at 0 °C for psychrophilic ones were additionally calculated. The ΔG at 37 °C for *Entamoeba histolytica* was −33.9064 kcal⋅mol^−1^, *Entamoeba dispar* −28.6764 kcal⋅mol^−1^, *Balamuthia mandrillaris* −34.4748 kcal⋅mol^−1^, and *Vermistella antarctica* −57.9398 kcal⋅mol^−1^ at 0 °C. The values of the ∆G of Amoebozoa are presented in Table 4.

### 3.4. Morphotypes and Free Binding Energy Values

**Figure 6:**
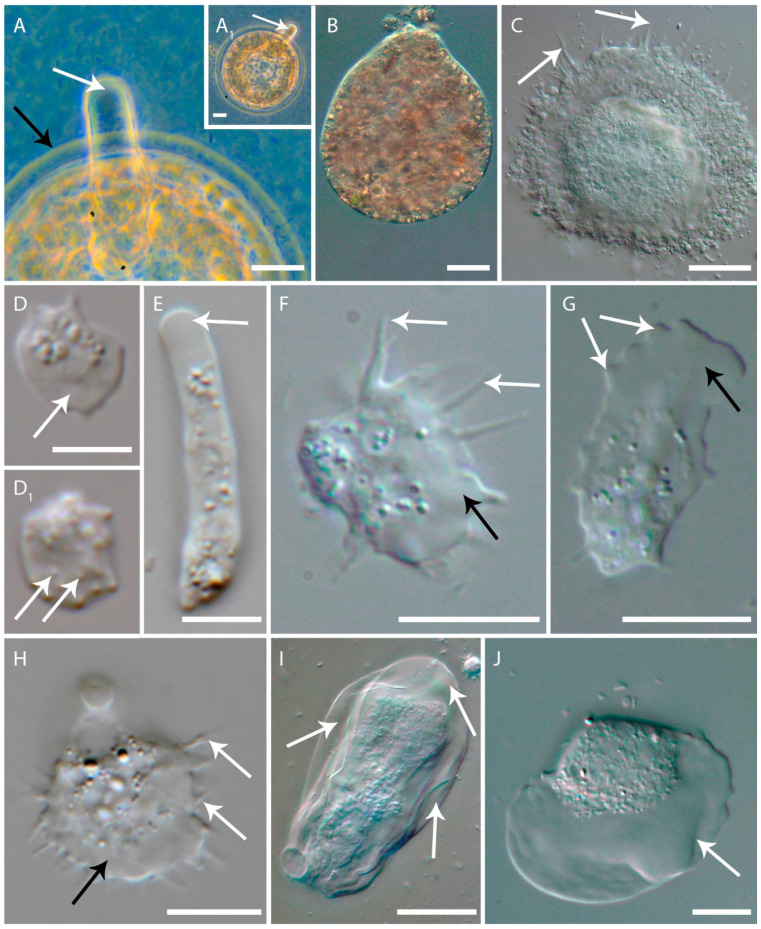
Locomotive forms of Amoebozoa species. (**A**)—cylindrical pseudopodium of *Arcella* sp.: white arrow shows the hyaline cap; black arrow shows the shell. (**A1**)—the view of the whole *Arcella* sp. cell: arrow shows pseudopodia. (**B**)—*Pelomyxa shciedti*. (**C**)—*Gocevia* sp.: arrows show subpseudpodia (hyaline projections). (**D**)—*Armaparvus* sp., view from the “dorsal” side: arrow shows hyaline area. (**D1**)—view from the “ventral” side: arrow shows subpseudopodia attached to the substrate. (**E**)—*Stygamoeba* sp.: arrow shows hyaline area. (**F**)—*Vexillifera abyssalis*: white arrows show hyaline projections (subpseudopodia); black arrow shows hyaline area. (**G**)—*Neoparamoeba aestuarina*: white arrows show hyaline projections (subpseudopodia); black arrow shows hyaline area. (**H**)—*Acanthamoeba castelliani*: white arrows show hyaline projections (subpseudopodia); black arrow shows hyaline area. (**I**)—*Thecamoeba* sp.: white arrows show hyaline area. (**J**)—*Vannella* sp.: white arrows show hyaline area.

For 20 species, whose sequences were included in the MD analyses, we analyzed the morphological characteristics of the locomotive forms (Table 5). To unify the hyaline cytoplasmic structures that amoebae may produce during locomotion, we distributed them between three groups: (1) hyaline area (lamellipodia-like, fan-shaped, or frontal-lateral hyaline area), (2) hyaline projections (filose pseudopodia, adhesive filaments, and subpsedopodia), and (3) hyaline cap at the tip of the pseudopodia. The hyaline area is typical for *Vannella*, *Clydonella*, and *Thecamoeba* (Figure 6I,J). Some species, such as *Neoparamoeba aestuarina*, *Acanthamoeba*, and *Vexillifera,* can produce both structures: the hyaline area and hyaline projections (Figure 6F–H). *Mastigella*, *Rhizomastix,* and *Gocevia* do not form hyaline area, but can produce a hyaline projection (Figure 6C, Table 5). The hyaline structures can take part in the directed cell motion or not. The citation of the descriptions is presented in Table 5. The species were ranked in decreasing order of the free binding energy values in Table 5. *Arcella intermedia* that produces pseudopodia with the hyaline caps showed the highest ∆G value, −28.6353 (kcal⋅mol^−1^) (Figure 6A, Table 4). *Pelomyxa schiediti* does not produce any hyaline formations and also has a high ∆G value, −34.527 (kcal⋅mol^−1^) (Table 4). *Vexillifera abyssalis* shows medium ∆G values and produces hyaline projections as well as the hyaline area (Figure 6F, Table 4). Mainly, as the ∆G values decrease, the presence of the hyaline area becomes predominant, while the presence of hyaline protrusions decreases (Figure 6, Table 4). However, there is an exception to the rule; for example *Rhizomastix vacuolata* produces hyaline projections only and have a ∆G value higher than *V. abyssalis* (Figure 6F, Table 4 and Table 5). Thus, *Vannella* and *Vermistella* with the lowest ∆G value lower than *V. abyssalis* demonstrate the hyaline area without protrusions comprising more than ½ of the cell length (Figure 6J, Table 4 and Table 5).

### 3.5. Statistical Analyses of Morphology and ΔG

For statistical analyses, we discarded most of Dictyostelids, since the morphological descriptions of their myxamoebas is insufficient. According to the morphological features of Amoebozoa described in the literature, we split the species into four groups: A (no formation of hyaloplasm), B (hyaline projections), C (hyaline projections and hyaline area), and D (hyaline area) (Table 5 and Table 6). The one-way ANOVA showed that there was a difference between the morphological groups. The F-value was 17.614, the critical F-value was 3.169, and the p-value was 1.36625·10^−5^ with α=0.05. The Tukey test showed that there were significant differences between the A–C, A–D, B–D, and C–D pairs of groups and there were no significant differences between A–B and B–C.

## 4. Discussion

### 4.1. Actin-Binding Sites

The actin-binding sites in Arp2 and Arp3 were described for *S*. *pombe* [31]. Our analysis of the same positions in Amoebozoan sequences revealed one identical position for Arp2 and three positions for Arp3. We can suggest here that these identical positions are basic for the stability of the Arp2/3 complex with actin. It is remarkable that there are three identical positions for all Amoebozoa and *S. pombe* in Arp3, while there is only one identical position in Arp2. We can hypothesize that this may be due to the fact that Arp3 is the first subunit that interacts with the first monomer of actin [31]. Therefore, we can suggest that identical positions are required for the stability of the first interaction. Other amino acids that are crucial for the interaction of Arp2 and Arp3 with actin can vary depending on the group. Apparently, these positions may be modified in different groups because they are less involved in the stability of the complex.

### 4.2. 3D Models of Arp2 and Arp3 Interactions and Insertions

We designed for the first time the 3D models of the interacting subunits of Arp2 and Arp3 for Amoebozoa. The 3D models are very similar for all Amoebozoa, except of *Arcella intermedia* that lost the region taking part in the interaction between subunits Arp2 and Arp3. We can suggest that this deletion affects the ΔG of the subunits in *A. intermedia*.

The interaction between subunits is maintained mainly by the van der Waals surface complementarity of amino acid residues. There are solvent-inaccessible zones between the overlapping residues. They also provide a contribution to the interactions between subunits. It is remarkable that there are conservative positions of amino acid residues—i.e., Ile (186)-Asn (203), whose contribution in the energy of binding varied in different species despite the invariability of residues. The surface complementarity varied within 0.55–0.85, and the buried SASA (the fraction of the solvent-accessible surface area of the residue that is buried by the interaction with residues in the other group) varied within 78.7–89.2%. This variation may depend on the configuration of each subunit separately and the configuration of the bond complex that is determined by the total contribution of all amino acids.

### 4.3. Energies and Morphology

We juxtaposed the calculated FBE of species with the morphological features of the locomotive form and revealed a pattern associated with the hyaloplasm zone in locomotive forms. For those species where a clear description of the locomotive forms with the micrographs is available, we found, next, orderliness. The lowest ΔG = −58.8 kcal⋅mol^−1^ of Arp2 to Apr3 connection was shown for Vannellida, the amoebae with the broad hyaline area that occupies half of the cell body [4]. The highest ΔG was calculated for *Arcella intermedia* that have only a hyaline cap at the tips of the pseudopodia [42,61] (Figure 1). We noticed a tendency in the series of ΔG values. In this series, amoebae forming wide anteriolateral hyaline area have the lowest ΔG: Vannelida, *Thecamoeba*, and *Vermistella*. The amoebae that are able to form thin hyaline projections along with a hyaline area had intermediate values of the free binding energy, i.e., *Armaparvus* sp., *Vexillifera*, *Neoparamoeba*, and *Acanthamoeba* (Table 5, Figure 6D,D1,F–H). The next group with higher ΔG values containing *Gocevia*, *Mastigella*, and *Rhizomastix* shows the tendency to lose the hyaline area (Table 5, Figure 6C). *Mastigella* have some wide formations of hyaloplasm, while *Gocevia* sp. have thin hyaline projections. And, finally, *Arcella* and *Pelomyxa* lost the hyaline formation except a small hyaline cap at the tips of the pseudopodia in *Arcella* (Figure 6A). *Endolimax* also belongs to this group. However, the morphological data for this genus are insufficient. Nevertheless, there are drawings of *Endolimax nana* in the old literature that show no hyaline projections [44,45]. We should mention here that the division between the morphological groups is based on only a single morphological characteristic—hyaloplasmic structures. Thus, some species turned out to be exceptions for their groups such as *Stygamoeba regulata* in group D. *Stygamoeba regulata* as well as *Vermistella antarctica* have very similar locomotive forms, elongated with a frontal hyaline area, and have close ΔG values at 27 °C [49,59]. However, *Vermistella antarctica* is a psychrophilic species and adopts locomotive forms only at 0 °C [59], while, in S. *regulata,* is a non-psychrophilic species [49]. Therefore, for the ANOVA analysis, the temperature values where amoebae demonstrated their activities were taken into account. The ANOVA showed that morphological group D (amoebae with hyaline area) differed significantly from all other morphological groups. In turn, group A (no hyalaloplasm) differed significantly from groups C and D that possess a hyaline area (Table 6). Thus, we can suggest that the formation of an anteriolateral hyaline area without any other hyaline structures likely requires lower values of the free binding energy. In contrast, the absence of any hyaline formations corresponds to the highest values of ΔG. According to thermodynamic relationships, the extent of the protein–ligand association is determined by the magnitude of the negative ΔG. Therefore, ΔG may determine the stability of any given protein–ligand complex (Du et al., 2016). A lower ΔG for the inactive protein complex indicates a more challenging transition between inactive and active forms of the complex. Thus, a lower free binding energy of the inactive protein complex results in a more stable protein–protein connection. We can suggest that, at a low ΔG, the Arp2/3 complex will have a more difficult transition from the inactive to the active form. As a result, the actin network will be likely more stable. Apparently, a stable actin network is necessary in order to maintain the frontolateral hyaline area permanently during the directed movement of the cell involving the Arp2/3 complex as seen in Vannellida. On the contrary, amoebae that do not form pronounced hyaline structures, such as species of *Arcella intermedia* or *Pelomyxa schiedti*, exhibit high ΔG values. This suggests that the energy-consuming process of transitioning between inactive and active bonded Arp2 and Arp3 is low for these species, leading to a low stability in the complex of bonded subunits for *Arcella intermedia* or *Pelomyxa schiedti*. Since the Arp2/3 complex is an actin-binding protein, it provides the formation of the structures that are maintained by actin nets, such as lamellipodia [62,63,64]. The anteriolateral hyaline area is morphologically very similar to lamellipodia, and is also supported by the actin filaments [5,6,65]. Interestingly, *Entamoeba hystolitica* and *E*. *dispar* also have high ΔG values despite a very specific locomotion that is more similar to the locomotion of *Nolandella* sp. or *Coronamoeba villafranca* [22,66,67]. However, the ability of *E*. *dispar* and *E*. *hystolitica* to form so-called lamellipodia and filopodia in contact with a chemoattractant like fibronectin was recently shown [22,68,69]. Unlike in, for example, vannellids, these hyaline structures are not constantly produced in *Entamoeba* during locomotion. We did not a reveal statistically significant difference between morphologically close groups: A (no hyaline structures) and B (hyaline projections), and B and C (hyaline projections and hyaline area). The absence of significant differences between these groups may be caused by the insufficient sampling. For example, one of the abundantly presented groups in databases of sequences is Dictyostelida. However, the very scarce morphological data of dictyostellid myxamoebae forced us to discard most of the dictyostelids from the statistical analyses. The inclusion of the new data in the statistical analyses will show statistical differences between close groups.

## 5. Conclusions

In conclusion, we predicted 3D models of the Arp2 and Arp3 subunits for 30 Amoebozoan species based on the 3D models of the Arp2 and Arp3 subunits of Schizosaccharomyces pombe. We determine the regions of interaction between subunits Arp2 and Arp3 and the amino acid residues that involved in the interaction. The taxon-specific and conservative amino acid residues interacting between the subunits Arp2 and Arp3 were determined. Finally, the binding free energy for the Arp2–Arp3 bond was calculated for 28 Amoebozoan species. There is a dependency between the binding free energy value and the hyaloplasm zone in the moving amoebae.

## Figures and Tables

**Table 1:** The crucial amino acids for interaction of actin with Arp2 and Arp3 in *Schizosaccharomyces pombe* [31].

Protein	Subdomain	Domain	AA
Arp3	3	W-loop (Y200)	Y200, L179, A166
Actin (n)	2	D-loop	M44
Arp3	1	-	L393 Y156 Y402
Actin(n)	2	D-loop	Val45
Arp2	3	W-loop (V169)	V169, Y143
Actin (n + 1)	2	D-loop	M44
Arp2	1	-	I360, M367
Actin (n + 1)	2	D-loop	Val45

**Table 2:** Crucial amino acids for *S. pombe* and their substitutions in Amoebozoan sequences.

Protein	*S. pombe* S6W17 Position	*D. discoideum* Position	Residue	Region	Substitutions in Amoebozoan Sequences
ARP2	V169	Y171	Var	Mod var	Y or F. C: *Entamoeba*
ARP2	Y143	Y145	Ident	Cons	Y
ARP2	I360	LI362	Cons	Mod var	L: *Dictyostellida* and *Arcella*. V: *Entamoeba* and *Thecamoeba quadrillineata*. I: the rest of Amoebozoa
ARP2	M367	F370	Cons	Mod var	F: Amoebozoa. A: *Entamoeba*.
ARP3	Y200	Y184	Ident	Cons	Y
ARP3	L179	L163	Ident	Cons	L
ARP3	A166	A151	Cons	Cons	A: Amoebozoa. V: *Entamoeba*.
ARP3	L393	L384	Cons	Cons	L: Amoebozoa. M: *Entamoeba*
ARP3	Y156	Y141	Ident	Cons	Y
ARP3	Y402	V393	Var	Var	L: *Acytostellium* and *Heterostellium*. A: *Paramoeba*, *Mastigamoeba*, *Endolimax*. H: *Entamoeba*. I: *Tieghemostelium lacteum*. V: the rest of Amoebozoa.

**Table 3:** Amino acid residues of Arp2 and Arp3 interacted between Arp2 and Arp3 of *D. discoideum* and some characteristics of their interactions.

Residue in Arp3	Closest Residue in Arp2	Distance Between Residues	vdW Surface Complementarity	Buried SASA
118:Leu	201:Ala 248:Arg	4.4 A 5.0 A	0.47	50.5%
119:Asn			0.00	3.6%
120:Ala			0.00	0.3%
121:Pro	200:Tyr 199:Gly 198:Arg	3.4 A 4.7 A 5.4 A	0.54	76.6%
124:Arg	199:Gly 200:Tyr 201:Ala	3.0 A 5.2 A 5.2 A	0.60	57.5%
144:Val			0.00	20.1%
181:Ser	203:Asn 204:Arg	4.7 A 5.0 A	0.35	47.3%
182:Glu			0.00	0.4%
184:Tyr	204:Arg 196:Leu 203:Asn 202:Phe	3.8 A 4.3 A 5.2 A 5.2 A	0.50	63.7%
185:Val	201:Ala 202:Phe 203:Asn	4.1 A 5.2 A 5.3 A	0.36	81.2%
186:Ile	203:Asn	3.6 A	0.55	88.2%
187:Gly	201:Ala	5.1 A	0.00	20.2%
188:Ser	206:Ala 207:Asp 205:Thr 203:Asn	3.7 A 4.4 A 5.1 A 5.4 A	0.88	56.3%
190:Ile			0.00	15.0%
306:Ser	64:Ser	5.3 A	0.00	17.7%
308:Pro	205:Thr	3.8 A	0.00	35.2%
309:Ile	67:Gln	4.4 A	0.00	40.5%
311:Cys			0.00	3.0%
345:Tyr	41:Ser	4.8 A	0.14	8.7%
346:Arg			0.00	1.6%
349:Arg	43:Glu 41:Ser	3.3 A 3.9 A	0.31	32.6%
350:Ser	40:Arg 41:Ser	4.0 A 5.1 A	0.25	34.4%
353:Leu	41:Ser 40:Arg 38:Ile	3.2 A 3.5 A 4.8 A	0.87	59.4%
354:Ser	40:Arg	3.5 A	0.84	20.7%
409:Arg	198:Arg 199:Gly 200:Tyr 251:Lys	2.9 A 4.0 A 4.2 A 5.4 A	0.80	62.0%
410:Phe	197:Leu 199:Gly 198:Arg 196:Leu	3.3 A 3.4 A 3.5 A 4.4 A	0.89	57.9%
417:Ile	193:Lys	5.2 A	0.00	14.5%

**Table 4:** The ΔG (kcal·mol^−1^) of amoebozoan species at 27 °C.

Species, GenBank Accession No. for Arp3 and Arp2, Respectively	Clade	∆G (kcal·mol^−1^)	Standard Deviation	Standard Error
*Arcella intermedia* GIBP01003616, GIBP01003406	Tibulinea	−28.6353	5.966	0.8437
*Cavenderia fasciculata* XP_004359239, XP_004360449	Dictyostellida	−31.9235	5.2496	0.7424
*Endolimax* sp. GKKX01057543, GKKX01051299	Archamoeba	−33.0986	3.2762	0.4633
*Pelomyxa schiedti* KAH3742643, KAH3745257	Archamoeba	−34.527	5.3595	0.758
*Acytostelium subglobosum* XP_012759837, XM_012897186	Dictyostellida	−34.6123	4.9998	0.7071
*Polysphondylium pallidum* XM_020577421, XM_020573619	Dictyostellida	−35.6981	4.449	0.6292
*Mastigella eilhardi* DAQZSK010023081, DAQZSK010023278	Archamoeba	−35.721	7.0651	0.9992
*Mastigamoeba abducta* DAQZSJ010084483, DAQZSJ010006647	Archamoeba	−37.5266	5.8399	0.8259
*Gocevia fonbrunei* GELP01001215, GELP01001322	Centramoebida	−38.4316	5.1354	0.7263
*Hagiwaraea rhizopodium* GIOY01006717, GIOY01027751	Dictyostellida	−38.5619	4.8208	0.6818
*Physarum polycephalum* GDRG01007989, GDRG01008543	Myxogastria	−38.6009	5.5212	0.7808
*Armaparvus* sp. HBSP01005665, HBSP01015409	Cutosea	−38.9269	4.9057	0.6938
*Balamuthia mandrillaris* GISS01000877, LEOU01000276	Centramoebida	−39.5976 −34.4748 *	7.3995 6.3267 *	1.0465 0.8947 *
*Stygamoeba regulate* HBLF01032544, HBLF01005862	Stygamoebida	−41.4434	6.433	0.9098
*Vexillifera abyssalis* HBYB01010238, HBYB01021282	Dactylopodida	−42.752	5.9331	0.8391
*Heterostelium album XP*_020432649, *XP*_020435691	Dictyostellida	−43.0832	5.6217	0.795
*Tieghemostelium lacteum* KYQ96802, KYR01241	Dictyostellida	−43.1193	6.019	0.8512
*Rhizomastix vacuolata* GKKW01043818, GKKW01014206	Archamoebida	−43.6755	4.7774	0.6756
*Entamoeba dispar* *Entamoeba dispar* XP_001740530б XP_00173478	Archamoebida	−43.176 −28.6764	5.5247 5.3734	0.781 0.7599
*Dictyostelium discoideum* XP_638880, XP_645275.	Dictyostellida	−44.9985	5.3929	0.7627
*Paramoeba aestuarina* HBKR01004080, HBKR01040389	Dactylopodida	−47.127	8.3094	1.1751
*Acanthamoeba castellanii* XP_004351625, XP_004353426	Centramoebida	−47.9059	7.9741	1.1277
*Thecamoeba quadrilineata* GELS01000580, GELS01000438	Thecamoebida	−49.3666	7.0039	0.9905
*Entamoeba histolytica* BAN38591, BAN39721	Archamoebida	−51.6515 −33.9064 *	5.7372 7.9704 *	0.8114 1.1272 *
*Vannella* sp. uncultured HBXS01014348, HBXS01011182	Vannellida	−52.3999	4.7277	0.6686
*Clydonella* sp. ATCC 50,884 GELR01000729, GELR01000581	Vannellida	−56.6988	7.457	1.0546
*Vermistella antarctica* GELU01002683, GELU01002815	Stygamoebida	−42.6115 −57.9398 **	7.799 7.1130 **	1.1029 1.0059 **
*Vannella* sp. KDN1 IACZ01000199, IACZ01001928	Vannellida	−58.8438	5.8405	0.826

* For Balamuthia mandrillaris, Entamoeba hystolitica, and Entamoeba dispar, the ΔG values at 37 °C. ** For Vermistella Antarctica, the ΔG values at 0 °C.

**Table 5:** Morphological features of the locomotive forms with the description citation and references. The list of species is ranked in decreasing order of the free binding energy values.

Species Name	Description Citation	Reference	Hyaloplasmic Structures, Participation in Motility (yes/no)
*Arcella* sp.	Tubular pseudopodia with the hyaline cap at the tips.	[42]	Hyaline cap, no
*Pelomyxa schiedti* KAH3742643	Eruptive anterior lobopodia.	[43]	-, no
*Mastigella eilhardi* DAQZSK010023081	A few lobate pseudopodia.	[43]	Hyaline projections, no
*Mastigamoeba abducta* DAQZSJ010084483	The anterior part of the cell was hyaline. Some gliding cells produced fine pseudopodia from the anterior and posterior part of the cell. The aflagellated cells moved slowly with eruptive hyaline lobopodia.	[44]	Hyaline area, hyaline projections, no
*Gocevia fonbrunei* GELP01001215	--	-	Hyaline projections
*Physarum polycephalum*	Actin-enriched cytoplasmic domain arises during the amoeboflagellate transformation. The ridge is a well-defined cytoplasmic domain that comprises up to approximately 25% of the projection area of cells imaged.	[45]	Hyaline projection *
*Armaparvus languidus*	Filose projections or adhesive filaments.	[46]	Hyaline projections, hyaline area **, yes
*Balamuthia mandrillaris* GISS01000877	Lobose pseudopods, or by spider-like activity of numerous radiating non-branching pseudopods. Hyaloplasm was localized at the tip of the leading pseudopodium and extended to nearly 1/7 of the total cell length.	[47,48]	Hyaline cap, no
*Stygamoeba regulata* HBLF01005862	The anterior hyaloplasm was pronounced and occupied some 1/4 to 1/3 of the cell length, but rarely more.	[49]	Hyaline area, yes
*Vexillifera abyssalis*	Flattened anterior hyaline area that was semicircular or fan-shaped; narrow hyaline subpseudopodia with the length equal to the length of the cell.	[50]	Hyaline area, hyaline projections, yes
*Rhizomastix vacuolata* GKKW01043818	Crawling cells formed small pseudopodia.	[51]	Hyaline projections, yes
*Entamoeba dispar*	Prominent lamellipodia 13% Small lamellipodia 41% Filopodia 10% Combination of filopodia and lamellipodia 36% (percent out of all cytoplasmic formations)	[22]	Hyaline area, hyaline projections, yes
*Dictyostelium discoideum* XP_638880.1	Lamellipodium (actin-rich), hyaline projection.	[45,52,53]	Hyaline area, yes
*Paramoeba aestuarina*, HBKR01004080	In the moving cells, hyaloplasm occupied 1/2–3/4 of the cell body.	[54]	Hyaline area, hyaline projections, yes
*Thecamoeba quadrilineata* GELS01000580	*Thecamoeba* spp., hyaloplasm ordinarily an anterolateral crescent.	[55]	Hyaline area, yes
*Entamoeba histolytica*	Prominent lamellipodia 66% Small lamellipodia 26% Filopodia 1% Combination of filopodia and lamellipodia 7% (percent out of all cytoplasmic formations)	[22]	Hyaline area, hyaline projections, yes
*Clydonella* sp.	Hyaloplasm occupies half or more of the total body area.	[56,57,58]	Hyaline area, yes
*Vermistella antarctica* GELU01002683	There is no comprehensive description. According to the light microscopic micrographs, there are hyaline subpsedopodia more than ½ of the cell length.	[59]	Hyaline area, yes
*Vannella* sp. IACZ01000199	A wide frontal area of the hyaloplasm.	[4,21,60]	Hyaline area, yes

* Despite the fact that this formation looks wide, it is not frontal and does not take a part in the motility. ** Despite the presence of a hyaline area, *Armaparvus* sp. has a very specific type of movement that is rather similar to Pellita, and a very small size of cell, 5–10 µm (7 µm).

**Table 6:** Morphological groups of species for one-Way ANOVA. The groups were split by the type of hyaloplasm formations.

A (No Hyaloplasm Formation)	B (Hyaline Projections)	C (Hyaline Projections and Hyaline Area)	D (Hyaline Area)
*Arcella intermedia*	*Mastigella eilhardi*	*Mastigamoeba abducta*	*Stygamoeba regulate*
*Endolimax* sp.	*Gocevia fonbrunei*	*Vexillifera abyssalis*	*Dictyostelium discoideum*
*Pelomyxa schiedti*	*Physarum polycephalum*	*Paramoeba aestuarina*	*Thecamoeba quadrilineata*
*Balamuthia mandrillaris*	*Rhizomastix vacuolata*	*Acanthamoeba castellanii*	*Vannella* sp. uncultured
*Entamoeba histolytica*		*Armaparvus* sp.	*Clydonella* sp. ATCC 50884
*Entamoeba dispar*			*Vermistella antarctica*
			*Vannella* sp. KDN1

## Data Availability

All data are available in the Supplementary to this article.

## Supplementary Materials

The following supporting information can be downloaded at: https://www.mdpi.com/article/10.3390/biom14121583/s1, S1—S1.fasta. Full alignment of 79 sequences of Arp3 of Amebozoa. S2—S2.fasta. Full alignment of 98 sequences of Arp2 of Amebozoa. S3—S3.zip. PDB files with 3D structures of unbound and connected subunits Arp2 and Arp3 in inactive form. File names: prefix_A.pdb—Arp3; prefix_B.pdb—Arp2; prefix_com.pdb—bond subunits. Prefix—genus name. S4—S4.zip. Tables of interactions of Arp2 and Arp3 residues. File names: genus name _table.csv. Table S1—Table_S1.docs. Table S2—Table_S2.docs.
