# Supplementary material for: From Molecules to Amoeboid Movement: A New Way for Understanding the Morphology Through Actin-Binding Proteins
Source: Biomolecules. 2024 Dec 11;14(12):1583. doi: 10.3390/biom14121583 (PMC11673790; doi:10.3390/biom14121583)
Supplement: Supplementary file 1 [file biomolecules-14-01583-s001.zip › Table_S1.pdf]

Table S1. Identical and conserved amino acid residues in the sequences of Arp2 and Arp3

Arp2	
**Position**	**Residue**
1	M
7-8	(hyd)(hyd)
10-17	DNGTGFVK
19-21	GFA
24-26	NFP
29-33	(hyd)FPSM
35-40	GRP(hyd)(hyd)R
42-45	EEK(hyd)
50-54	(hyd)KD(hyd)M
56-59	GDEA
62-63	(hyd)R
70-72	YPL
74-80	NG(hyd)(hyd)(pos)(pol)W
82-83%	D(hyd)
86-87	(hyd)W
89	Y
91	(hyd)
93-95	E(pos)L
105-109	(hyd)(hyd)LTE
111	P
113-114	NP
117-118	N(pos)
121	M
125-127	MFE
131	F
135-136	Y(hyd)
138-142	(hyd)QAVL
144-167	LYAQG(hyd)(hyd)TGVVVDS GDGVTH(hyd)(hyd)PV
170	G
176	L
179-180	RL
182-184	(hyd)AG
186-188	D(hyd)T
191-192	LI
194-196	L(hyd)L
198-200	RGY
202-212	FNRTADFET(hyd)R
214-217	(hyd)KE(pos)
219-220	CY
223-224	(aro)D
228	E
231	L
234-236	ETT

Arp3	
**Position**	**Residue**
1	M
7-26	LPAV(hyd)(hyd)DNGTGYTK(hyd)G (aro)AGN
28-30	(neg)P(pol)
33-35	(hyd)P(pol)
37-38	IA
57-60	D(hyd)DF
62-63	IG
65-66	EA
73	Y
78-79	P(hyd)
82-83	GQ
86-88	(pol)WT
90-91	ME
94-95	WE
98-103	(hyd)F(pos)Y(hyd)R
106	P
108-119	DH(aro)(hyd)LLTEPP(hyd)N
121-125	PENRE
128-155	AE(hyd)(hyd)FE(pol)FNVPG(hyd)YI AVQAVLA(hyd)(hyd)A(hyd)W(pol)
163-180	LTGTV(hyd)DSG(neg)GVTH(hyd)IP (hyd)
182-188	(neg)GYVIGS
190-191	IK
193-198	IP(hyd)AG(pos)
200-201	(hyd)(pol)
204	(hyd)
206-207	(pol)(hyd)
210-211	(aro)R
215-216	(hyd)P
224	A
228-229	KE
234	V
237-238	D(hyd)
240-241	(pos)E
246	D
262	(hyd)
270-274	D(hyd)GYE
276-277	FL
279-280	PE
282-287	FF(pol)PEI
289-291	(pol)SD
294-296	(pol)PL
299-301	(hyd)VD
305	Q

Arp2	
238-239	L(hyd)
244-253	LPDGR(hyd)(hyd)(pos)(hyd)G
255-256	ER
259	A
261	E
263-264	(hyd)F
266	P
275	G
280-281	(hyd)F
284-285	I(pol)
290	D
295-296	(hyd)Y
298-307	H(hyd)(hyd)LSGG(pol)(pol)M
309-315	PGLPSR(hyd)
317-319	K(neg)(hyd)
322-323	LY
341-353	RIEDPPRRKH(hyd)VF
355	GGAVLA
362	(hyd)
365-366	D(pos)
369-371	(hyd)W(hyd)
373	(pos)
375	E
378	E
380	G
386	K

Arp3	
307-310	CPID
312-313	RR
315-329	LYKNI(hyd)LSGGSTMF(pos)
331	F
334-341	RL(pol)RD(hyd)(pos)(pos)
344	D
346	R
353-354	LS
356-357	G(pos)
365	V
367-370	V(hyd)(pol)(aro)
373-374	QR
376-378	AVW
380-385	GGS(hyd)(hyd)(hyd)
388	P
390	F
394-397	CH(pol)K
400	Y
402	E
404	G
407	I
409	R
411	N
413	V

Uppercase letters are identical residues
Lowercase letters in brackets are conservative residues that have the following properties:
hyd -- hydrophobic; pos -- positive charge; neg -- negative charge; pol -- polar; aro -- aromatic
