# Supplementary material for: From Molecules to Amoeboid Movement: A New Way for Understanding the Morphology Through Actin-Binding Proteins
Source: Biomolecules. 2024 Dec 11;14(12):1583. doi: 10.3390/biom14121583 (PMC11673790; doi:10.3390/biom14121583)
Supplement: Supplementary file 1 [file biomolecules-14-01583-s001.zip › Table_S2.pdf]

Supplementary Table S2. Amino acid residues of Arp3 and Arp2 subunits interacting via vdW complementarity in *D. discoideum* and differences with other Amoebozoan species.

**Residue of Arp3**		**Residue of Arp2**		**vdW surface complementarity range in amoebozoan species**	**Buried SASA range in amoebozoan species**
**Changes in species in this position: Residue (Species name)**	**D. discoideum**	**D. discoideum**	**Changes in other species in this position: Residue (Species name)**		
Met (Entam)	Leu (118)	Ala (201)	Val (Acanth) Tyr (Clydo, Vann) Ser (Entam)	0.14-0.79	43.9-58.9
		Arg (248)			
	Pro (121)	Tyr (200)		0.26-0.70	71.2-82.3
		Gly (199)	-- (Verm)		
		Arg (198)	-- (Acanth, Acyt, Balam, Clydo, Dict_p, Gocev, Heter, Phys, Rhizo, Styga, Vann, Vann_u, Verm)		
	Arg (124)	Gly (199)		0.30-0.77	55.7-97.1
		Tyr (200)	-- (Vermi)		
		Ala (201)	Val (Acanth); Tyr (Clydo, Vann) Ser (Entam) -- (Vermi)		
Ala (for all but Caven, Dict)	Ser (181)	Asn (203)		0.23-0.54	21.7-58.8
		Arg (204)	-- (Arcel)		
	Tyr (184)	Arg (204)		0.28-0.72	33.8-63.7
		Leu (196)			
		Asn (203)			
		Phe (202)	-- (Arcel, Clydo, Vann); Leu (Rhizo)		
	Val (185)	Ala (201)	Val (Acanth); Tyr (Clydo, Vann); Ser (Entam)	(0.20 Arcel) 0.31-0.60	75,7-93
		Phe (202)	Leu (Rhizo)		
		Asn (203)			
	Ile (186)	Asn (203)		0.55-0.85	78,7-89.2
	Ser (188)	Ala (206)		0.77-0.92	56.3
		Asp (207)			

		Thr (205)	-- (Vermi)<br>Ser (Vann)		
		Asn (203)	-- (Arcel, Clydo, Endol, Vermi)		
His (Clydo, Endol, Vexil);<br>Phe (Entam, Heter, Polys, Van_u);<br>Gln (Hagiw, M-eba);<br>Thr (M-lla)<br>Trp (Armap, Physa)<br>Leu (Raper)<br>Val (Theca)<br>Asn (Vermi)	Tyr (345)	Ser (41)	-- (Arcel);<br>Tyr (Balam, Gocev, Styg, Vexil);<br>Phe (Clydo, Param, Theca, Vann);<br>Thr (M-eba);Ala (Armap)	0-0.28<br>0.53 (Rhizo)<br>0.62 (M-eba)	1.5-14.7
Lys (Endol, Entam, Rhyzo, Van_u)<br>Asn (M-eba)	Arg (349)	Glu (37)	-- (Arcel, M-eba)	0.21-0.78	17.5 (M-eba)<br>18.6 (Armap)<br>29.4-46.5
		Ser (35)	Tyr (Balam, Gocev, Styg, Vermi, Vexil);<br>Phe (Clydo, Param, Theca, Vann);<br>Thr (M-eba)<br>Ala (Armap)		
-- (Arcel)<br>Thr (Endol, M-eba, Rhizo);Ala (Entam)	Ser (350)	Arg (40)	-- (Arcel, Balam)	0.13-0.92	19.9-73.7
		Ser (41)	Tyr (Balam, Gocev, Styg, Vexil);<br>Phe (Clydo, Param, Thecam, Vann);<br>Thr (M-eba)<br>-- (Armap, Physa)		
	Leu (353)	Ser (41)	Tyr (Balam, Gocev, Styg, Vermi);<br>Phe (Clydo, Theca);	0.40 (Endol)<br>0.82-0.93	42.4-69.4

			Thr (M-eba)<br>Ala (Armap)		
		Arg (40)			
		Ile (38)	-- (Balam, Clydo, Gocev, Param, Physa, Rhizo, Theca, Vann)*;<br>Leu (Entam, Van_u);<br>Met (M-eba)<br>Phe (Param, Vann)		
Thr (Endol, M-lla)	Ser (354)	Arg (40)		0.23 (Dict_p)<br>0.79-0.93	19.3-75.1
--<br>(Tieg)**	Arg (409)	Arg (198)	His (Physa)	0.60-0.84	56.4-75.2
		Gly (199)			
		Tyr (200)	-- (Tiegh, Vermi)		
		Lys (251)	-- (Acanth, Arcel, Dict_p, Endol, Ent_d, M-eba , Armap, Rhizo, Theca, Tiegh, Vermi, Vexil)<br>Tyr (Vann)		
--<br>(Tieg)**;<br>Tyr (Arcel, Caven, Endol, Entam, Hagiw, Raper, Theca)<br>His – the rest of species	Phe (410)	Leu (197)	Ile (Ent_h)<br>Val (Ent_d)	0.55-0. 90	28.9-67.5
		Gly (199)			
		Arg (198)	-- (Acanth, Tiegh)<br>His (Physa)		
		Leu (196)	Gln (Clydo, Vann)		

* In the absence of Ile (38), Glu (42 or 43) is present.
** In Arp3 of *T. lacteum*, Lys (443) and Tyr (448) aligned with Arg (409) and Phe (410) of *D. discoideum* but, the interaction with Arp2 was provided by amino acids Lys (467) and Arg (468).

Designations:
-- - The participation of the residue in the interaction was not recorded;
Amoebozoan species: Acanth – *Acanthamoeba castellanii*, Arcel – *Arcella intermedia*, Balam – *Balamuthia mandrillaris*, Caven – *Cavenderia fasciculata*, Clydo – *Clydonella sp.*, Dict_p – *Dictyostelium purpureum*, Dict – *Dictyostelium* any species, Endol – *Endolima* sp., Ent_d -- *Entamoeba dispar*, Ent_h -- *Entamoeba histolytica*, Entam -- *Entamoeba* any species, Gocev – *Gocevia fonbrunei*, Hagiw – *Hagiwaraea rhizopodium*, Heter – *Heterostelium album*, M-eba – *Mastigamoeba abducta*, M-lla

– *Mastigella eilhardi*, Param – *Paramoeba aestuarina*, Armap – *Armaparvus* sp., Physa – *Physarum polycephalum*, Polys – *Polysphondylium pallidum*, Raper – *Raperosteliu potamoides*, Rhizo – *Rhizomastix vacuolata*, Styga – *Stygamoeba regulate*, Theca – *Thecamoeba quadrilineata*, Tiegh – *Tieghemostelium lacteum*, Vann – *Vannella* any species, Van_u – *Vannella* sp. uncultured, Vermi – *Vermistella antarctica*, Vexil – *Vexillifera abyssalis*.
